# COVID-19: From health crises to food security anxiety and policy implications

**DOI:** 10.1007/s13280-020-01481-y

**Published:** 2021-02-19

**Authors:** Linley Chiwona-Karltun, Franklin Amuakwa-Mensah, Caroline Wamala-Larsson, Salome Amuakwa-Mensah, Assem Abu Hatab, Nolwandle Made, Nathan Kanuma Taremwa, Lemayon Melyoki, Lettice Kinunda Rutashobya, Thulisile Madonsela, Marna Lourens, Wendy Stone, Alfred R. Bizoza

**Affiliations:** 1 Swedish University of Agricultural Sciences, Box 7012, 75007 Uppsala, Sweden; 2 University of Gothenburg, Box 645, 405 30 Göteborg, Sweden; 3 Institute of Computer and Systems Sciences -SPIDER, DSV, Stockholm University, Postbox 7003, 164 07, Kista, Sweden; 4 Department of Business Administration, Technology and Social Sciences, Luleå University of Technology, 971 87 Luleå, Sweden; 5 College of Agriculture, Animal Sciences and Veterinary Medicine (CAVM), University of Rwanda, Kigali, Rwanda; 6 University of Dar es Salaam Business School, Dar es Salaam, Tanzania; 7 Faculty of Law Trust Chair in Social Justice, Stellenbosch University, Cape Town, South Africa; 8 Department of Economics & Rural Development, Arish University, Al-Arish, Egypt

**Keywords:** COVID-19, Feminist economics, Food security, Policy, Social protection, Sub-Saharan Africa

## Abstract

Like the rest of the world, African countries are reeling from the health, economic and social effects of COVID-19. The continent’s governments have responded by imposing rigorous lockdowns to limit the spread of the virus. The various lockdown measures are undermining food security, because stay at home orders have among others, threatened food production for a continent that relies heavily on agriculture as the bedrock of the economy. This article draws on quantitative data collected by the GeoPoll, and, from these data, assesses the effect of concern about the local spread and economic impact of COVID-19 on food worries. Qualitative data comprising 12 countries south of the Sahara reveal that lockdowns have created anxiety over food security as a health, economic and human rights/well-being issue. By applying a probit model, we find that concern about the local spread of COVID-19 and economic impact of the virus increases the probability of food worries. Governments have responded with various efforts to support the neediest. By evaluating the various policies rolled out we advocate for a feminist economics approach that necessitates greater use of data analytics to predict the likely impacts of intended regulatory relief responses during the recovery process and post-COVID-19.

## Introduction

When 2019 The State of Food Security and Nutrition Report (FAO 2019) chose the theme: safeguarding against economic slowdowns and downturns emphasizing the importance of protecting nutrition and food security during crisis, presciently it was preparing for the COVID-19 pandemic. At the time of writing, more than 1400 000 lives have been lost due to the pandemic globally and the numbers continue to rise (John Hopkins University 2020). Regulatory responses such as lockdowns have negatively undermined economic outcomes and stability while highlighting existing gross disparities, social injustices, availability and access to food and food security (Arndt et al. 2020). Disruptions in food supply chains cause limited availability and access to food (Reardon et al. 2020; Zhou 2020) especially where households are dependent on day-to-day purchases of fresh produce, particularly among the poor and vulnerable, the majority of whom are women (Devereux et al. 2020; Harris et al. 2020; Malapit et al. 2020). Countries with no nationwide welfare policies to enhance household food security (Banerjee et al. 2015) and that are heavily dependent on food imports are especially likely to suffer during crises and food disruptions (Vogel and Smith 2002). Several African countries experience some form of food insecurity, particularly among poor households and remote or rural communities. Women being largely in the informal sector in these countries tend to experience food insecurity due to vulnerability and not having much access to resources, networks or decision-making power (Quisumbing et al. 2015; Malapit et al. 2020). Other key drivers of food insecurity include poverty, conflict, climate change, disrupted ecosystem services and economic decline (Misselhorn 2005). A country’s ability to be food secure depends on its resource endowments, natural and cultural capital, and policies and the extent to which these resources are applied (Carletto et al. 2013).

The COVID-19 pandemic forced countries in Africa to undertake strong measures ranging from total lockdowns, partial lockdowns, to stay-at-home orders (SAHO) (Arndt et al. 2020). These prescriptions restricted mobility, livelihoods, gender equality and family support networks, leading to socioeconomic consequences, including major loss of income (Ragasa and Lambrecht 2020) and disruption of agriculture activities such as farming, access to farm inputs and markets (Arndt et al. 2020) that have exacerbated food insecurity and related nutrition deficiencies (Naja and Hamadeh 2020). Depending on the implementation of these measures and related policies to ease the effects of COVID-19 on a national basis, households may experience varying levels of concern for food and nutrition security, especially where urban–rural partnerships become severed (Sukhwani et al. 2020). The impact of food security deterioration under COVID-19 has worsened the abilities for compliance of African countries with the right to food as protected in article 25 of the Universal Declaration of Human Rights and related instruments (Mechlem 2004).

Almost one-fifth of Africa’s population (256 million) is undernourished, and the number of hungry people is increasing (FAO 2019). Africa’s population is predicted to double by 2050 and food demand to triple (United Nations 2020a, b, c). To feed the growing population and to ensure healthy lives and the promotion of well-being in line with the sustainable development goals (SDGs) especially 1 on poverty and 2 on food and health (United Nations General Assembly 2015) require conserving ecosystems while reducing inequalities (Fleetwood 2020). Many of the African countries that experience an increase in hunger during economic downturns are dependent on primary commodities for export and/or imports and the livelihoods of a substantial part of their population depend on food systems (SOFI 2019). The COVID-19 pandemic could double the number of acute crisis-level hungry people, increasing from 113 million to 265 million, including 73 million people in acute hunger crisis mode (Global Report on Food Crisis 2020). The pandemic has exacerbated food supply problems, with the transport and processing of food reducing the availability of basic food items (Arndt et al. 2020; Béné 2020; Devereux et al. 2020).

The poor and most vulnerable are often key players in these disrupted sectors, as they are engaged in tasks such as planting and harvest, transport, processing, in-country trading and distribution to and from local markets (Chiwona-Karltun et al. 1998; Lambert et al. 2020). Because of soaring unemployment and under-employment especially in the informal sector and urban areas, the decline in purchasing power also blocked access to sufficient food (Global Report on Food Crisis 2020). In addition, internal and cross-border mobility restrictions have caused economic and food accessibility challenges, given that remittances in 2018 accounted for US$46 billion compared to US$32 billion foreign direct investment in sub-Saharan Africa. Reduced remittances (which in 2018 accounted for US$46 billion compared to US$32 billion foreign direct investment in sub-Saharan Africa), restrictions in mobility and decline in employment formally and informally have curtailed the well-being and food security situation of households during the pandemic (Lambert et al. 2020). For example, the negative impact on informal financial remission channels poses a serious food security problem for migrants (Duvenage 2020; Madonsela et al. 2020). According to Lawson-Lartego and Cohen (2020), “if people are worried about what to eat and other basic needs, they will not respect the lockdowns necessary to combat the COVID-19 pandemic” reflecting a special nature of this COVID-19 shocks as compared to the 2002 food crisis (Vogel and Smith 2002), 2008 world financial crisis and other crisis (Schmidhuber and Qiao 2020).

The impacts of the wide array of lockdown measures are yet to be fully investigated (Kosnik and Bellas 2020), especially in Africa where agriculture is the backbone of the economy, livelihoods and household food security (Lawson-Lartego and Cohen 2020). The intersections of gender and age with food insecurity have gained even less attention, at this early stage in the investigations (Ragasa and Lambrecht 2020; Zeinali et al. 2020). Policy tracking studies by IFPRI on agriculture- and food security-specific responses to COVID-19 progress reveal little focus on women or gender balance (Ragasa and Lambrecht 2020). This will have grave implications given that women face different constraints from men (de Paz et al. 2020) as food producers, processors and traders. According to Ragasa and Lambrecht (2020), gender-sensitive agricultural programmes and interventions that are more inclusive reduce gender inequalities, including through gender-responsive policies (Quisumbing et al. 2015). Gender-responsive data produced in such a crisis can evaluate how gender-inclusive farming efforts affected community resilience, particularly in agrarian societies of Africa.

Thus, this paper evaluates the local spread and economic impact of COVID-19, and the implications of lockdown measures on food security in selected sub-Saharan Africa countries, using observational cases and quantitative digital online data sources. It contributes to the ongoing debate about the socioeconomic impacts of COVID-19 with focus on food security in Africa by providing quantitative estimates of the local spread and the economic impact of COVID-19 on food worries. Second, the paper illustrates some concrete examples of how specific countries have addressed issues pertaining to food security through measures related to social protection and social justice. Finally, the paper highlights how African governments have proactively applied some fiscal, monetary and economic measures to bridge income gaps and to ensure the well-being of its populations in response to immediate or mid- to long-term impacts of the pandemic. By evaluating the various policies rolled out, we advocate for a feminist economics approach that necessitates greater use of data analytics to predict the likely impact of intended regulatory relief responses during the recovery process and post-COVID-19.

## Feminist approach to food security

In building a case for a feminist economist theory approach to government interventions, we briefly consider two key terms that circulate within food studies, namely food security and food sovereignty. The latter concept draws parallels with feminist economic theory in advocating for the recognition of the individual/gendered-dimensions contributions to food production. Food sovereignty demands food justice in its understanding for subsistence farming as the base of commercial forms of agricultural production (Desai and Mies 2012). In other words, while commercial interests and global food interests may especially value commercial agriculture and its contribution to food security, a food sovereign context recognizes that subsistence farming is the foundation of commercial farming. Feminist economists similarly argue that women contribute more than 40% of the food that is consumed worldwide and yet their contribution does not receive commensurate value or recognition (Mies 2010). The same women while farming for the nutrition of their families also supply commercial production, highlighting their double contribution. Feminist economists argue for recognizing unpaid contributions to a nation’s economy, similar to what food sovereigntists suggest with subsistence farming.

On the flip side, food security as advocated by FAO (2011) is ensuring global food production that meets the nutrition and food preferences required to lead active and healthy lives, suggesting multitier levels of food security beyond safe and nutritious food (Sachs 2013). Access to food additionally empowers and dignifies individuals and their social relations. To that end, the discourse around food security hinges on four supportive pillars: availability, access, utilization and stability. In providing a feminist analysis of food security, we briefly define each pillar giving mention to the gender-differentiated needs with each pillar.

- *Availability* is measured by agricultural production and data feeding the overall view of food availability is on a macro, national level. Even though women are said to produce at least 40% of the world’s food (Sachs 2013), their limited access to resources required for production places them at a disadvantage when it comes to how much value their input contributes. As food activists would have it, some bodies are valued more highly than others for their contribution to food production (Mupotsa 2016).
- *Access* addresses direct correlations between women’s decision-making power and access to and control over resources, investment in the resources that ensure food availability and who then has access to food, be it physically, socially or economically (Richards et al. 2013). As with availability, women’s access to inputs vital for food production is limited, meaning that food access at household level involves gendered inequities to food access when women and girls are the ones without sufficient nutrition in lieu of other family members (Pilla and Dantas 2016).
- *Utilization* is addressed in connection with food access as the nutritional value of food consumed. Food security encompasses food quality, nutrition and safety. As women assume the bulk of food preparation in many contexts, their role in translating the food available to meet dietary and nutritious family needs also falls on them (FAO 2011). Malnutrition is rife in individuals where unequal gender relations limit the access of girls and women to nutritional knowledge, food and decision-making processes (Smith and Haddad 2015).
- *Stability* speaks to the reliability, consistency and stability of food availability, food access and food utilization. Questions around stability extend to economic/financial stability in food prices, the ability to store food and continued access to food even during times of crises such as the current pandemic (Coates et al. 2007). Women are more likely to experience cycles of anxiety in light of the food responsibilities that rest upon them as discussed above.

Approaches and policies to food security from a macro level may fail to adequately address the gender-differentiated needs, as the governance of dominant food systems may unintentionally fail to consider the granulated levels of food production discussed here. A feminist analysis of food security argues for the right of all women, men, girls and boys to have access to safe, healthy and adequate food. We take the practical feminist approach to ensuring the recognition of women’s central role in food security, advocating feminist economic theory (FET) as a lens through which food worries are scrutinized. FET stresses the value of understanding gender relations in food production, processing and trading and their contribution to global and national functioning of economics. Food activists argue in this regard for the recognition of men and women as economic agents equally contributing to food security (cf. Waring and Steinem 1988).

The tenets of FET have at their root the move to equitably improve the well-being of everyone regardless of gender, age, status or class. Hence as governments work on food security, distributional measures need to be socially just in valuing unpaid contributions from women. Our study employs a FET lens to further develop a deeper understanding of the impacts of COVID-19 on food security. More specifically, this article considers to what extent policy interventions are inclusive in counting unmeasured economic phenomena such as the informal economy, unpaid work and social collective drives, considering that most statistics bureaus draw on data from the market economy to inform national accounting policies. The feminist approach is particularly useful in untangling the disparities and the positions women, men and youth assume during crises such as COVID-19.

## Conceptual framework

The conceptual framework adopted in this study builds on complementary insights drawn from the FAO’s ‘four pillars approach’ as outlined above (FAO 2008), the ‘food systems’ approach (HLPE 2017) and Sen’s ‘entitlement’ approach (Sen 1981) for the analysis of food security impacts of COVID-19 (Devereux et al. 2020). Devereux et al. (2020) have also reflected on the potentials of these three frameworks in explaining food security impacts of COVID-19. They argue that the ‘four pillars’ approach (i.e. availability, access, utilization and stability) and the food systems approach (entails all elements and activities that relate to the environment, production, processing, distribution, preparation and consumption; and the socioeconomic and environmental outcomes) provide an umbrella covering both supply and demand factors at the aggregate level. Unlike these frameworks, the entitlement approach is suitable for disaggregating and nuancing the demand-side drivers of access to food at household level. It also highlights mechanisms such as informal transfers and social capital which are not well covered by the two other frameworks.

Despite the emergence of literature on the impacts of COVID-19 in Africa, case study reports and quantitative analysis assessing the combined effects of health and economic consequences of COVID-19 on food supply and food security outcomes are scarce. A few studies have been initiated in South Africa by the social justice and COVID-19 policy and monitoring alliance (SCOPRA) that aims to constitute a social accountability bulwark and regularly track COVID-19 policies and relief measures in line with SDG 10, 16 and 17. Our main hypothesis is that food security in Africa has been negatively affected by COVID-19 health and economic impacts and women are more vulnerable. Women are more negatively affected than men due to disruptions in production, demand and supply, especially in the informal sectors and trading which are dominated by and normally employ mostly women (Wamala-Larsson and Svensson 2018). Consequently, women are likely to be the most anxious—psychologically affected and concerned—physiologically about food security resulting from COVID-19 impacts.

Socioeconomic policies and their operationalization are foundationally skewed towards institutional/formal productive labours which to a great extent are the focus of national surveys that eventually drive national economic accounting practices. Informal labour practices that dominate most African economies are not adequately captured by national accounts. While women dominate the informal economy (Arndt et al. 2020), significant proportions of the sector are dominated by men such as informal transport services that serve the mobility needs of millions of Africans (Wills et al. 2020), and which are also at risk of food insecurity.

**Fig. 1:**
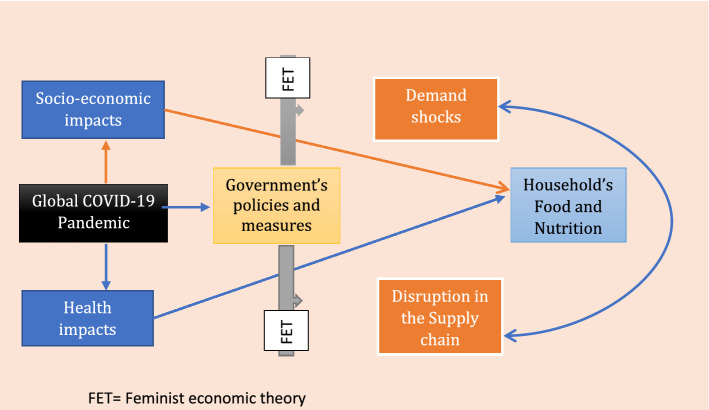
Impact pathways of COVID-19 on Household’s Food and Nutrition Security

In addition, where women may contribute labour, for example farming efforts that are remunerated, this contribution is undervalued (Chiwona-Karltun et al. 1998). Consequently recognizing and recounting the statistics that may inform resource distribution during pandemics such as this one are imperative. The conceptual framework in Fig. 1 proposes a feminist economic approach to household food security. Through FET, government policies would recognize the full spectrum of women’s paid and unpaid work, respecting its value as part of the process of promoting equal access to resources. Marilyn Waring and Steinem (1988) credited for a feminist reading of national income accounting suggests that standards measuring need may not be sufficiently sensitive to gender dimensions and data used to inform resource distribution may require redressing if it is to track economic trends. FET calls for a multi-stakeholder approach to national economics accounting and what nations must do to ensure equity in the distribution of resources during crises such as the COVID-19 pandemic.

## Methods and data

Our study relies on both qualitative and quantitative analysis. The qualitative analysis is based on documentation and description of government containment measures put in place in sub-Saharan Africa (SSA) countries in the period of the COVID-19 pandemic. The data for this part of the study are based on the ACAPS^1^ database, which combines all the measures implemented by governments worldwide in response to the COVID-19 pandemic. Our policy analysis focuses on government policies (Moorty et al. 2020) on food and economic support that selected SSA countries based on ACAPS database have implemented during the COVID-19 pandemic. In addition, we provide observational cases of events relating to food security and consideration of gender dimensions linked to the specific countries used in the quantitative analysis.

Based on the conceptual framework developed in Fig. 1, we modelled the effect of concerns about COVID-19 on food security. Our empirical model is specified as the probability of food worries as a function of the level of concern about COVID-19, socioeconomic and demographic variables and country fixed effect. The outcome variable is measured by whether the individual has been worried about not having enough food to eat in the past 7 days prior to the survey because of lack of money or other resources. The outcome variable $\left( y \right)$ is a binary variable which takes the value of 1 if an individual has been worried about not having food to eat, and 0 otherwise. Equation (1) shows the empirical model;

1 $$y_{ijt} = \beta_{0} + \beta_{1} Covid19\_Concern_{ijt} + \beta_{1} GovtPolicy_{j} + X_{ijt} \beta + \eta_{j} + \gamma_{t} + \varepsilon_{ijt}$$

where the subscripts $i, j and t$ represent the observation for each individual, country and survey wave, respectively; $Covid19\_Concern$ is a variable capturing the individual’s level of concern about (1) the local spread of COVID-19 and (2) economic impact of COVID-19. On a scale of 1 to 5, with 1 being not concerned and 5 being very concerned, the individual is asked to indicate their level of concern about the local spread (or economic impact) of COVID-19 in their respective country. $GovtPolicy$ is a categorical variable representing the various movement restriction measures implemented by the government to halt the spread of the virus. These include no lockdown, stay at home/curfew, partial and full lockdown. The vector $X_{i}$ represents socioeconomic and demographic factors which serve as control variables (such as gender, age, locality (that is, urban–rural), country fixed effect $\left( {\eta_{j} } \right)$ and survey wave-fixed effect $\left( {\gamma_{t} } \right)$. The random error term is captured as $\varepsilon_{ijt}$.

Given the feminist economics approach in our conceptual framework, coupled with demographic distribution of COVID-19 infection cases and fatalities rate, our study considers subsample analysis across gender and age groups. In addition, issues about food security and COVID-19 infection differ across urban and rural areas, so we also consider subsample analysis focusing on these areas. As governments’ movement restriction measures in controlling the spread of the virus differ across countries, we expect heterogeneous effects on the level of concern about COVID-19 on food worries; hence, we also investigate this by restricting the sample by restriction measures.

As the outcome variable is binary, Eq. (1) is estimated by using a probit model and we report the marginal effects in this study. The standard errors in our estimations are clustered at the primary administrative unit to account for possible correlation in the residual of the outcome variable (food worries) among individuals within the same community. Food price is a key determinant of demand which could also affect whether an individual worries about food (Ryan et al. 2020). Our dataset does not have food price information and other relevant factors, which may affect food worries and could correlate among individuals. By clustering the standard errors at the community level, we adjust the standard errors for inference. Amuakwa-Mensah et al. (2020) have used a similar approach.

In addition to the data limitations mentioned above, individual information such as employment status of the individual is not captured in our dataset. Furthermore, the data used in this study do not have household information about the respondent such as household size, household dependency ratio, income and housing characteristics, which may affect food security. Where FET would laud the survey research for extracting data from the individual as a unit of analysis as opposed to the household as an economic unit for understanding food security worries, FET would still ask the extent to which binding social relations were measured (cf. MacDonald 1995). FET in this case would want to know who the data represent and the accuracy of its depiction of reality, assuming that these variables would affect the extent to which the local spread of COVID-19 is likely to increase their worry about not having food to eat.

Our study approaches the “food worries” categories as socially constructed phenomena. This enables us to critically evaluate their development, appropriation and acceptance as driven by human interests and as such understand practices in and around them as socioculturally influenced. We are therefore aware of the contextual diversity of these categories and in our analysis and conclusions draw partial generalizations. Approaching gender, age, locality and restrictions as social constructions can improve the objectivity of practice around food security. Drawing on FET, we push for untangling the gendered processes in productive and reproductive labour, specifically the valuation of women’s unpaid work. Through FET, we scrutinize the distribution of income and resources as they pertain to realizing food security as we attempt to understand the people behind the statistics.

The quantitative analysis relies on an open-access survey dataset that was designed and collected by GeoPoll.^2^ Two rounds of the survey were administered through SMS and mobile web. The first round occurred between 2 April and 9 April 2020, and the second round from 24 April to 8 May 2020. The data comprise 12 sub-Saharan Africa countries: Benin, the Democratic Republic of Congo, Ghana, Ivory Coast, Kenya, Mozambique, Nigeria, Rwanda, South Africa, Tanzania, Uganda and Zambia. A total sample of 4788 was collected across the 12 countries in Africa for the first round and about 4037 individuals were sampled in the second round. A simple random sampling technique was used by GeoPoll to select respondents from their database, which consists of a list of mobile subscribers in each country surveyed. The sample was roughly nationally representative by age, gender and location.

The descriptive statistics of the variables for the quantitative analysis are shown in Table 1. About 45% of the sample are females and 55% are males. The average age is around 31 years and 67% of the samples live in urban areas. Approximately, 80% of the sample indicated that they have been worried about not having enough food to eat in the past 7 days prior to the survey due to lack of money or other resources. Most individuals were concerned about both the local spread of COVID-19 and economic impact of the virus, with an average score of 4.26 and 4.29, respectively.

**Table 1:** Descriptive statistics of variables for analysis

Variable	Number of observation	Mean value	Standard deviation	Min	Max
Food worries	8782	0.803	0.398	0	1
Female	8782	0.447	0.497	0	1
Urban	8782	0.669	0.471	0	1
Level of concern COVID-19	8782	4.259	1.367	1	5
COVID-19 economic impact concern	8782	4.293	1.287	1	5
Age	8782	30.83	9.91	15	91

Food worries	Yes^a^	No^b^
Gender		
Female	0.446	0.451
Male	0.554	0.549
Locality		
Urban	0.660	0.706
Rural	0.340	0.294
Age groups		
Age ≤ 35	0.724	0.660
35 < age < 60	0.265	0.313
Age ≥ 60	0.106	0.277
Restrictions		
Full lockdown	0.724	0.276
Partial lockdown	0.817	0.183
Stay at home/curfew	0.812	0.188
No lockdown	0.738	0.263

*Source* Authors’ own construction from GeoPoll data. Partial lockdown: DR Congo, Ivory Coast, Ghana, Nigeria, Rwanda, Uganda. Lockdown: South Africa. Stay at home/Curfew: Benin, Zambia, Kenya, Mozambique. No lock: Tanzania

^a^Implies individuals indicated they worry about food

^b^Means individuals do not worry about food

## Results and discussion

Tables 2, 3, 4, and 5 report the results of our four-dimensional analysis of the effect of concern about COVID-19 on food security. In all the estimated models, we accounted for country- and wave-fixed effects. The Wald’s Chi-square and Pseudo R square statistics indicate the goodness of fit of the models. The following subsections discuss the marginal effects of each dimension of the analysis.

**Table 2:** Marginal effect of probit estimation of concern about local spread of COVID-19 on food worries

Variables	(1)	(2)	(3)	(4)	(5)	(6)	(7)	(8)
	Full sample	Female	Male	Age ≤ 35	35 < Age < 60	Age ≥ 60	Rural	Urban
COVID-19 concern	0.015***	0.015**	0.014***	0.016***	0.012*	0.008	0.009	0.018***
	(0.004)	(0.006)	(0.004)	(0.004)	(0.006)	(0.027)	(0.006)	(0.005)
Restrictions (ref: No lockdown)								
Stay at home/Curfew	0.040*	− 0.014	0.087***	0.034	0.059		0.025	0.046*
	(0.022)	(0.031)	(0.033)	(0.023)	(0.041)		(0.034)	(0.024)
Partial Lockdown	0.112***	0.061**	0.163***	0.090***	0.156***	0.153	0.104***	0.115***
	(0.030)	(0.028)	(0.054)	(0.035)	(0.058)	(0.243)	(0.038)	(0.039)
Lockdown	− 0.011	− 0.044	0.019	0.018	− 0.027	− 0.079	0.029	− 0.037
	(0.026)	(0.033)	(0.040)	(0.027)	(0.047)	(0.244)	(0.037)	(0.035)
Observations	8782	3925	4857	6248	2411	110	2908	5874
Country FE	YES	YES	YES	YES	YES	YES	YES	YES
Wave FE	YES	YES	YES	YES	YES	YES	YES	YES
Pseudo *R*^2^	0.0291	0.0329	0.0300	0.0171	0.0454	0.208	0.0220	0.0351
Wald chi2	187.3	78.62	97.12	157.4	114.4	41.29	58.06	136
Diff. Test COVID-19 concern (chi2)	0.04	0.57						
Diff. Test Partial Lockdown (*χ*^2^)	3.52*	0.81		0.0004				

The outcome variable is whether or not the individual has been worried about not having enough food to eat in the past 7 days prior to the survey because of lack of money or other resources. The outcome variable is a binary variable. We controlled for covariates such as gender, age, age squares, locality, wave and country fixed effect in all the models. Standard errors are clustered at primary administrative unit

Robust standard errors in parentheses. ***p < 0.01, **p < 0.05, *p < 0.1

**Table 3:** Marginal effect of probit estimation of concern about economic impact of COVID-19 on food worries

Variables	(1)	(2)	(3)	(4)	(5)	(6)	(7)	8))
	Full sample	Female	Male	Age ≤ 35	35 < Age < 60	Age ≥ 60	Rural	Urban
COVID-19 economic impact concern	0.025***	0.028***	0.023***	0.028***	0.016**	0.024	0.020***	0.028***
	(0.004)	(0.005)	(0.004)	(0.004)	(0.006)	(0.043)	(0.005)	(0.005)
Observations	8782	3925	4857	6248	2411	110	2908	5874
Country FE	YES	YES	YES	YES	YES	YES	YES	YES
Wave FE	YES	YES	YES	YES	YES	YES	YES	YES
Pseudo R2	0.0336	0.0388	0.0336	0.0239	0.0463	0.210	0.0263	0.0391
Wald chi2	229.2	93.46	104.9	210.5	113.8	39.79	88.45	150.8
Diff. Test COVID-19 economic concern (*χ*^2^)		0.67		4.35**			0.73	

The outcome variable is whether or not the individual has been worried about not having enough food to eat in the past 7 days prior to the survey because of lack of money or other resources. The outcome variable is a binary variable. We controlled for covariates such as gender, age, age squares, locality, government restriction, wave and country fixed effect in all the models. Standard errors are clustered at primary administrative unit

Robust standard errors in parentheses. ***p < 0.01, **p < 0.05, *p < 0.1

**Table 4:** Marginal effect of probit models based on restriction (local spread of COVID-19)

Variables	(1)	(2)	(3)	(4)
	No lockdown	Stay at home/Curfew	Partial Lockdown	Lockdown
COVID-19 concern	0.003	0.009	0.016***	0.041***
	(0.016)	(0.006)	(0.005)	(0.011)
Observations	400	3200	4382	800
Country FE	YES	YES	YES	YES
Wave FE	YES	YES	YES	YES
Pseudo *R*^2^	0.0132	0.00989	0.0328	0.0801
Wald *χ*^2^	18.40	24.37	141.5	341.7
Diff. Test (*χ*^2^)			2.12	

The outcome variable is whether or not the individual has been worried about not having enough food to eat in the past 7 days prior to the survey because of lack of money or other resources. The outcome variable is a binary variable. We controlled for covariates such as gender, age, age squares, locality, wave and country fixed effect in all the models. Standard errors are clustered at primary administrative unit

Robust standard errors in parentheses. ***p < 0.01, **p < 0.05, *p < 0.1

**Table 5:** Marginal effect of probit models based on restriction (COVID-19 economic impact)

Variables	(1)	(2)	(3)	(4)
	No lockdown	Stay at home/Curfew	Partial Lockdown	Lockdown
COVID-19 economic impact concern	− 0.006	0.023***	0.026***	0.048***
	(0.019)	(0.007)	(0.004)	(0.016)
Observations	400	3200	4382	800
Country FE	YES	YES	YES	YES
Wave FE	YES	YES	YES	YES
Pseudo *R*^2^	0.0133	0.0146	0.0384	0.0822
Wald *χ*^2^	12.84	35.39	198.3	299.2
Diff. Test (2) vs (3)		1.5		
Diff. Test (3) vs (4)			1.06	

The outcome variable is whether or not the individual has been worried about not having enough food to eat in the past 7 days prior to the survey because of lack of money or other resources. The outcome variable is a binary variable. We controlled for covariates such as gender, age, age squares, locality, wave and country fixed effect in all the models. Standard errors are clustered at primary administrative unit

Robust standard errors in parentheses. ***p < 0.01, **p < 0.05, *p < 0.1

### Concerns about local spread of COVID-19 and worriedness about food security

The full sample results (Model 1) in Table 2 reveal that an individual’s concern about the local spread of COVID-19 significantly increases the probability of worrying about not having enough food to eat by about 1.5%. This finding supports previous research findings on the impact of pandemics and outbreaks on different dimensions of food security (e.g. Oyefara 2007; Rich and Wanyoike 2010; Hassouneh et al. 2012). According to this evidence, disease outbreaks and pandemics threaten production (Ohadike 1981) and access to food mainly through losses of income and assets, which consequently inhibit their ability to buy food. Poorer households in developing countries spend up to 80% of their income on food (Bashir and Schilizzi 2013) and have limited access to financial markets, making their food security particularly vulnerable to extreme events, economic downturns, and unpredictable income. In particular, a large proportion of SSA population is employed in the informal sector where they are self-employed (e.g. daily labourers, landfill waste pickers and street hawkers Schenck et al. 2018) or waged employees, and they are uncovered by health or unemployment insurance schemes. Furthermore, very few countries have national social welfare systems that safeguard the well-being of its citizens.

The results show that stay-at-home orders (SAHO) and partial lockdowns that SSA governments implemented as measures to prevent the spread of the virus were associated with amplified food security worries (Arndt et al. 2020). Several recent studies have found that mobility restrictions and social distancing measures due to COVID-19 have further triggered consumers’ worries about food insecurity, reflected in hoarding behaviours, panic-buying and stockpiling of groceries and rapid changes in food consumption habits and diets (Coopi 2020; FEWSNET 2020; Sukhwani et al. 2020; Pulighe and Lupia 2020). It is not surprising that individuals hoarded food given the demonstrated association between food, nutrition and health, especially in boosting the immune systems and reducing susceptibility to infection (Naja and Hamadeh 2020). Notably, the results also revealed that the ‘full lockdown’ was the only measure that had an insignificant effect on individual’s worries about not having food to eat, which could be attributed to two reasons. First, as the strictest COVID-19 containment measure employed to flatten the curve of incidence rate, which governments adopt to prevent the spread of the pandemic from the hotspots to other regions, few SSA countries implemented this measure compared to those that implemented partial and SAHO. Second, the major challenge of lockdowns in various countries has been its enforcement, as the degree of enforcement and use of sanctions to punish violators depend on the extent to which the policy is socially acceptable or the government is trusted, as well as government’s capacity to monitor and police compliance (Mboera et al. 2020).

With regard to gender differences, the results of Models 2 and 3 in Table 2 show that the sign and magnitude of the effect of COVID-19 concerns on perceived food insecurity worries by females and males are qualitatively similar and are in congruence with those of the full sample model. This finding is analogous to the general trend in the emerging literature on the impacts of COVID-19, which suggest that the pandemic often affect men and women differently (World Bank 2020) and found higher vulnerability among women in terms of both livelihoods (Ragasa and Lambrecht 2020) and dietary effects (Arndt et al. 2020). However, a comparison of the marginal effects of the restriction measures’ variables for males and females indicates that SAHO and partial lockdown measures significantly increased males’ perceived food insecurity worries compared to their female counterparts. There may be numerous reasons for this, the obvious one being the sample and how representative it may be of women’s food security worry; we recall that the female ratio of responses is 45%. Second, how the data are collected and analysed is a profoundly gendered process, in that women struggle to verbalize their own value contributions when asked, because they do not have the required vocabulary and they fail to see their own contributions to food security as real work (MacDonald 1995, p. 163). Statistical bureaus are therefore faced with the challenge of measuring and valuing unpaid work, when the standards of measurement are the market economy. FET is interested in understanding the interaction between paid economies and unpaid work especially as unpaid/informal work is embedded in measuring the market economy. This becomes all the more crucial when the economy is shut down even partially, because the measure of need may obscure critical voices.

In terms of heterogeneity across age groups, the results of models 4, 5 and 6 show a positive and statistically significant effect for COVID-19 concerns on individuals below the age of 36 years and those belonging to the age group from 36 to 59 years, with no significant difference between these two age groups. In contrast, the effect was statistically insignificant for older individuals over 60 years. Likewise, the variables related to restriction measures documented similar patterns of impact across age groups, where partial lockdown, the only measure with a statistically significant effect, was associated with increased worries about not having to eat among the individuals belonging to the age groups below 59 years. These conclusions are not surprising, considering Africa’s young population with a median age below 20 years. These findings correlate to the findings of FAO which indicated that the adverse effects of the COVID-19 on poverty and food security are uneven and more intense for younger individuals in the working age who due to the situation have been unable to generate income and do not have access to social protection networks (FAO-CELAC 2020). This raises concerns around issues of food quality and nutrition (Naja and Hamadeh 2020), socioeconomic status, and risk of metabolic diseases compounded by obesity due to consumption of cheap, highly processed energy-rich foods. These types of foods are readily consumed by lower socioeconomic groups (Clemmensen et al. 2020) and more affordable as has been observed in South Africa (Temple et al. 2011; Arndt et al. 2020).

From a spatial dimension, the results of models 7 and 8 reveal that concerns about the spread of COVID-19 significantly increase worries about food security among urban dwellers, whereas this effect was statistically insignificant in rural areas. This finding agrees with the findings of a recent OECD (2020) study showing that the impact of COVID-19 has a strong contextual dimension, while urban areas with their dense international links (e.g. international markets, business travel, tourism) are often the entry points for the virus and have been particularly hard hit. In the same context, Abu Hatab et al. (2020) attribute the spatial heterogeneity of the impacts of COVID-19 between rural and urban areas to population density, which is traditionally greater in urban centres of African countries. So are enforcements of governments’ containment measures, which are stricter with negative to violent repercussions if disobeyed, in African cities and towns. We reiterate the social constructedness of the “food-worry” categories and ask the extent to which spatial dimensions contribute to constructing decisions about the survey research design, the sampling techniques, the data collection process, the coding, analysis and the eventual decisions that are made from the data that would inform relief measures.

### Concerns about economic impacts of COVID-19 and worriedness about food security

Table 3 presents the marginal effect of the impact of the individuals’ concern about the economic impact of COVID-19 on their worries about not having enough food because of lack of money or other resources. Overall, the results further support our principal findings in the previous section (Table 2) and imply that the “economic impacts” of the virus have significantly raised individuals’ worries about food insecurity. Notably, the results reveal that the magnitude of the economic effect of COVID-19 is much higher than that of the concerns about the local spread of the virus, which is expected because the level of concern is a ‘feeling’ of worry and care and is influenced by personal identity, and a variety of interrelated demographic, political and cultural factors. Mapping together the results in Tables 2 and 3, our findings imply that the COVID-19 pandemic has triggered significant slow down and rapid contractions of the economic activity in SSA countries. These economic impacts presented the pathway through which the virus threatened individuals’ food security and raised their worries about not having enough food to eat. Our findings align with those of Thurlow (2020) showing that most COVID-19 impacts on the food systems are indirect and mainly caused by falling incomes and other economic consequences. In the same context, Udmale et al. (2020) argue that the COVID-19 pandemic presents an unprecedented public health crisis with significant economic repercussions that threaten food production and have the potential to result in food security crises in developing countries. Clearly, the COVID-19 pandemic is having a significant impact on food security in SSA.

The results show that the concern over the economic impact of the virus on food worries is relatively higher among females than males, though the difference is statistically insignificant. According to a United Nations’ report (2020a, b, c), “the economic impacts are felt poignantly by women and girls who are more often than not employed in the informal sector, generally earning less, saving less and holding insecure jobs or living close to poverty”. Similarly, Arnold et al. (2020) indicate that the economic shocks of the pandemic will have ripple effects on girls who are pulled out of schools to help at home or to work to support their families. FAO-WFP (2020) illustrates that females are likely to be disproportionately affected both in their productive roles (as producers, processors and traders) and in their reproductive roles and care functions, due to their time poverty and limited access to productive resources, services, information and decision-making power (Malapit et al. 2020). Emerging data are showing that children pulled out of school due to the lockdowns experience hunger and become more vulnerable for exploitation. For example, girls are more likely to encounter sextortion, unwanted pregnancy and various forms of gender-based violence (Rafaeli and Hutchinson 2020). Therefore, the pandemic may worsen existing gender-based violence and social inequalities, but also undermine future restoration efforts and food systems altogether.

The age group analysis in Table 3 shows a positive and statistically significant effect of concern about the economic impact of COVID-19 on food worries for individuals below 36 years and those from 36 to 59 years old, but not for those 60 and above. The effect is significantly higher for those below 36 years. This is because those from 36 to 59 tend to have relatively stronger job security or access to land ownership compared to those below 36 years.

Unlike the effect of the concern about the local spread of the virus on food worries, we find a positive effect of concern about the economic impact of the virus on food worries for both rural and urban areas. With regard to urban areas, COVID-19 has expanded in many cities to a crisis of urban equity, urban finance, safety, joblessness or labour layoffs, public services, infrastructure and transport, all of which are disproportionately affecting the most vulnerable in society (UN 2020b). In rural areas of developing countries, the ability to produce and consume one’s own food may be somewhat protective of food and diets during extreme events (Sukhwani et al. 2020). However, poverty and informal employment rates are generally higher in rural areas, whereas access to social networks is relatively lower, which combines to create a situation of extreme vulnerability (FAO-CELAC 2020). Despite the results pointing out that the effect of the economic impacts of COVID-19 on food worries is higher in urban than in rural areas, the results of our difference test indicate statistically insignificant difference between the two areas. FET suggests “adding on, or counting to complete the picture of what is needed to produce social well-being”, imploring for alternative views to how economies are envisioned. The contribution of informality and social collectives as enacted in different contexts are valued as part of the market economy (Cameron and Gibson-Graham 2003, p. 145). Envisioning these contributions has an impact on national interventions for food security.

### Restrictions, food worries and COVID-19 concerns

We examine the effect of concern about COVID-19 on food worries for the various government restrictions in Tables 4 and 5. From Table 4, we find that concern about the local spread of COVID-19 significantly increases the likelihood of food worries in countries with partial and full lockdown. An increase in the level of concern about the local spread of the virus increases the probability of individuals worrying about not having food to eat by 1.6% and 4.1% in countries with partial and full lockdown, respectively. Although the effect is higher for individuals who experienced full lockdown compared with those with partial lockdown, this difference is not statistically significant. In the case of concern about the economic impact of COVID-19 on food worries, we find a significantly positive effect among individuals in countries with stay at home/curfew, partial and full lockdown government measures. This effect increases with the level of the restrictions on movement. A marginal increase in the level of concern about the economic impact of the virus increases the probability of food worries by 2.3%, 2.6% and 4.1% among individuals living in countries with stay at home/curfew, partial and full lockdown government measures (see Table 5). We observed no statistical difference in the effect across the type of restrictions.

The positive effect of concern about COVID-19 on food worries due to the movement restriction could be explained by the impact of movement restriction on household income and physical access to food. As most individuals in SSA are employed in the informal sector and self-employed workers (e.g. daily labourers and street traders), the effect of the lockdown on these individuals implies a sudden drop in their income to zero hence worry about not having food to eat. Cases of panic-buying and hoarding food were reported during the period of restricted movement in the urban areas of some countries like Ghana, Nigeria, South Africa and Ivory Coast (KPMG 2020a, b. c), which resulted in exorbitant increase in prices. Coupled with the closure of open-air markets and a ban on street vendors in most countries during this period, this disrupted food access and reduced diet quantity and quality (Devereux et al. 2020). The restrictions also affected food production as farmers were unable to access farm inputs such as fertilizers and improved seeds due to restriction on movement and closure of geographic borders.

### Government relief policies during COVID-19

Table 6 depicts a summary of government initiatives and policies to mitigate the spread of COVID-19 and its consequences. We have identified and classified policy actions into three subthemes and related them with the four FAO pillars of food security. The purpose was to examine the likelihood of these actions contributing to *food production and availability* (such as social protection activities in kind, cash transfers, agricultural inputs and solidarity funds), *food access* (like the absorption of water and electricity bills by the government (Population Services International 2020); support to the informal sector; expediting unemployment benefits), *food stability* (enabling borders to be open such as the East African Community; implementing price controls on basic foods; managing food hoarding practices by dismantling lockdowns; accelerated response in conflict and climate disaster prone areas) and *utilization* (distributing food parcels to meet dietary needs; nutrition education awareness on quality of food distributed or bought; urban–rural-migration of families; women and children at risk; skipping of meals).

**Table 6:** Government population responses in mitigating effects of COVID-19 on food and nutrition security

Country/subthemes	Date of the First COVID-19 case	Social protection (in kind, food, and cash, agricultural inputs, solidarity fund or initiative)	Taxes, water and electricity and bills relief measures	Credit facility, removal money transaction and digital charges, economic recovery facilities	Sanitation and hygiene
Benin	16-03-2020	X	X	X	X
Democratic Republic of Congo (DRC)	10-03-2020	X			X
Ghana	12-03-2020	X	X	X	X
Ivory Coast	11-03-2020	X	X	X	X
Kenya	12-03-2020			X	X
Mozambique	22-03-2020	X			X
Nigeria	27-03-2020	X	X	X	X
Rwanda	14-03-2020	X	X	X	X
South Africa	05-03-2020	X	X	X	X
Tanzania	16-03-2020			X	X
Uganda	22-03-2020	X	X	X	
Zambia	22-03-2020	X		X	X

*Source* Authors’ compilation based on ACAPS database (https://data.humdata.org/dataset/acaps-covid19-government-measures-dataset) and other references in the text

The various responses though not necessarily coordinated seem to have taken into account the food and agricultural interconnections between countries beyond geographic borders. For example, food within the East African community freely moved between countries based on competitive advantages (IIED 2020). Similarly, food parcels were distributed in countries such as South Africa (Pais et al. 2020; Wills et al. 2020), taking into account that single-headed households would be most hard hit with 55% of population living below the poverty line (Kholer and Borhat 2020; Pietermaritzburg Economic Justice 2020; Parliamentary Monitoring Group 2020). While the food parcels may not have been fresh food but dry rations, tinned foods or processed foods, nutrition was to some degree a focus. Lockdowns during COVID-19 have affected the access to quality foods and healthy diets (Arndt et al. 2020; McCain 2020). Households with low levels of education and high dependence on labour income experienced real income shocks that jeopardized household food and nutrition security (Arndt et al. 2020). Having social protection measures did not sustainably safeguard against food insecurity during the “total lockdowns” especially for learners (Pikoli 2020), leaving households little choice but to opt for seeking income generation rather than avoid getting COVID-19 infection as has been observed with the HIV and AIDS crisis (Bryceson and Fonseca (2006).

Even in conflict prone and climate disaster areas such as Mozambique, interventions took into account measures that protect vulnerable groups (International Growth Centre (2020), particular attention was paid to food and nutrition security issues (Relief Web 2020). The establishment of a solidarity fund in South Africa with specific earmarked funding for agriculture vouchers for individuals involved in subsistence farming to ensure that farming continued (Solidarity Fund 2020). The provision of unemployment benefits in the informal sector in Ivory Coast safeguarded food stability and access to food at household level (KPMG 2020a, b. c; Garda World 2020). In Tanzania where the measures focused more on the economy, women in the informal sector, *mama ntilies* (informal food vendors operating at construction sites and office premises), did not receive much support, hence negatively affecting food security at household level (United Republic of Tanzania 2020). In some instances, men resorted as a coping mechanism in sending their families back home to the rural areas—resulting in families deconstructing. Lastly, several countries responded by ensuring that households could access food by providing relief through honouring water and electric bills, tax relief as well as provision of agricultural inputs and removal of collateral for loans (Deloitte and Touch 2020; World Bank 2020; Lawson-Lartego and Cohen 2020). However, despite these initiatives, effects of the pandemic are not yet robustly identified. Governments are not sure which policies would effectively constrain the effects of COVID-19. The research community is yet to provide comprehensive evidence and proposals regarding the COVID-19 particularly where women and gender inequalities abound. This is a clarion call for more sex-disaggregated data collection on COVID-19 economic and social effects in the context of food security.

## Conclusion and way forward

This paper has sought to discover how COVID-19 and related government restrictions have affected households’ food security in Africa. It has also sought to assess the adequacy of socioeconomic relief and related mitigation strategies. The article applies feminist economic theory to assess the differential impacts of the pandemic on women and gender dimensions likely to be overlooked in ongoing interventions by the government and development partners. We have used both quantitative and qualitative approaches to understand the data and to assess the effects of COVID-19 on food security in twelve countries from the four corners of Africa. The findings in this study have documented different impacts of COVID-19 for men and women and for urban and rural areas. These impacts have increased the burden on African countries in terms of securing food security both as a health and as an economic imperative but also as a matter of solidarity and human rights (South African Government 2020). The governments of the countries assessed here have proactively acted to contain the spread of COVID-19 and started initiating the economic recovery processes of the respective countries though one still observes increasing cases of COVID-19. Despite efforts made towards safe and stable nations, some constraining structural challenges hamper fully benefitting from the existing regional economic mechanisms such as the East African Community (EAC), Common Market for Eastern and Southern Africa (COMESA) and Southern African Development Community (SADC), among others. Except for a few attempts such as the Feed Africa Response (Blanke 2020), there is insufficient evidence of inter-state collaboration through the above economic regional bodies to collectively address the cross-border challenges relating to food security and to have collective solutions (Torero 2020). This is also summarized as 10 recommendations for African governments to ensure food security for poor and vulnerable populations during COVID-19 in a recent publication (Lawson-Lartego and Cohen 2020).

A major lesson from the government interventions during COVID-19 is that countries that had already established good social protection structures such as South Africa (Jansen 2020) and Rwanda (World Health Organization 2020) found it relatively easy to adapt quickly in decentralizing social protection interventions. Some cases of selective targeting and exclusion of social protection delivery have included leveraging food and financial relief for political gain. Another setback has been unconscionable price inflation and hoarding of food supplies and personal protection equipment (PPEs) by the private sector. We nevertheless observe a shift towards heightened food security awareness and responsiveness, but more effort is required to improve policy responses and governance (Maher et al. 2020).

If COVID-19 programmes had incorporated a feminist lens, better outcomes could have been tracked and achieved, with regard to both gender equity and broader social inclusion for all groups. This would have required using data analytics to predict the likely impact of intended regulatory and relief responses. The impact of this approach lies in the leveraging of gender disaggregated data and data disaggregated in other ways to predict the likelihood of a disparate social impact. This could apply to any future policy either in response to a pandemic or an everyday issue. Debates between economics and sociology have led to an understanding of the social embeddedness of individuals, advocating for a socioeconomic profile of the economy and its behaviour. FET and socioeconomists have a similar agenda in ensuring gender-aware practices in labour economics, household economics and socioeconomics (Fullbrook 2001). Counting food worries among men and women as individuals may fail to capture the full spectrum of social relationships in which the man or woman is embedded. In most African societies, these social relationships are the welfare system available to the men and/or women in disparate ways. Counting individuals as the only units of economic analysis ignores gendered institutions such as the household (cf Folbre 1994), meaning that if this is the data used to coordinate relief during pandemics, it may ignore more vulnerable and margnalized groups. FET would insist on accounting for the inequities in societies by combining quantitative methods with qualitative data to further the understanding of informal and unpaid work as vital parts of any economic system (Power 2004, pp. 4–5). FET argues that “unpaid work is an economic category and is endogenous to the economic process, for example in relation to labour supply” (van Staveren 2010, p. 26). As pointed out by Ragasa and Lambrecht (2020), much of the COVID-19 gender research published hitherto is an extrapolation for past crises.

Given that the right to food is a fundamental human right, it is also important that governments in Africa and elsewhere begin their policy design processes with an understanding that whatever they do should not undermine food security. To the extent that they seek to do so, they need to have a gender-aware compensation strategy before policies are passed and enacted.

It is also important that the recovery process looks beyond economic recovery to deal with all aspects of food security as specified in the four pillars of the FAO framework. Governments should deal with food security as a goal on its own (CIRAD 2020), not as a by-product of economic recovery. The recovery strategy should be anchored in the broader SDG agenda, including the advancement of equality in terms of SDG 5 and 10 and ending poverty and hunger in terms of SDG 1 and 2, respectively, while incorporating a feminist economic perspective. When Waring first issued her arguments against mainstream econometrics, she argued for better representation of informal, unpaid and care economies and that such work cannot be counted as one variable, but needs to be understood as a collective, cumulative over time, contribution to the base of the economy (Waring 1988).

## Footnotes

1 https://data.humdata.org/dataset/acaps-COVID-19-government-measures-dataset.

2 GeoPoll dataset can be accessed from https://data.humdata.org/dataset/covid-19-impacts-africa. The survey instrument can also be obtained from the same website. The dataset for both waves was directly requested from GeoPoll.
